# Symptoms in smokers trying to quit

**DOI:** 10.1186/1617-9625-3-2-44

**Published:** 2006-08-15

**Authors:** Tanja Tomson, Mats Toftgård, Hans Gilljam, Asgeir R Helgason

**Affiliations:** 1 Centre for Public Health, Stockholm County Council & Department of Public Health Sciences, Karolinska Institutet, Sweden; 2 Department of Oncology-Pathology, Karolinska Institutet, Sweden

## Abstract

**Aims:**

To describe the prevalence and intensity of different symptoms in relation to tobacco abstinence. To explore latent dimensions between symptoms in smokers trying to quit.

**Design:**

A cross sectional study using a questionnaire to retrospectively assess symptoms over a period of 12 months.

**Setting:**

Swedish telephone quitline, a nationwide free of charge service.

**Participants:**

All 741 individuals who had called the quitline and signed up for smoking cessation treatment between February 2000 to November 2001 and reported to have been smoke free for at least 24 hours during the previous 12 month period from first contact.

**Measurements:**

Assessments were made by self-report, and abstinence was defined as "not a single puff of smoke during the last week". A factor analysis approach where individual items aggregate into factors was used to explore the relationship between the different symptoms.

**Findings:**

High intensity of symptoms related to unsuccessful quitting attempts and included craving, irritability, apprehension/anxiety, difficulties concentrating, restlessness, depression/depressed mood, and insomnia. The factor loadings of all 17 symptoms resulted in three factors with factor 1, psychological being the most important. High scores on this factor relates to unsuccessful quitting attempts. Using Nicotine Replacement Therapy (NRT) for 5 weeks or longer, reduced symptoms included in factor 1. The other two factors were factor 2 physiological and factor 3 neurological.

**Conclusion:**

Symptoms that are psychological and/or neurological in nature are interrelated and appear to be the most significant obstacles for successful quitting attempts in a population-based setting. These symptoms may be successfully treated with NRT.

## Introduction

Many who try to quit smoking relapse within the first week [1,2] and 90 percent who attempt to stop smoking without support, relapse within one year [3,4]. Although most of our knowledge stems from closely monitored clinical drug trials, the understanding of why a majority of attempts to stop smoking fail, is incomplete.

One factor that may explain a failed quit attempt is the smoker's experience of nicotine withdrawal. Early relapsing smokers are thought to be more severely dependent, and smokers unable to stay quit have reported more severe withdrawal symptoms [5,6].

Although studies have focused on different symptoms and varying definitions of severity, they are generally consistent with the American Psychiatric Association Diagnostic and the Statistical Manual definition of nicotine withdrawal DSM-IV [7]. Nicotine withdrawal includes symptoms of anxiety/apprehension, restlessness, difficulty concentrating, depression/depressed mood, irritability, sleepiness/drowsiness, and insomnia [7]. Despite omission from the DSM-IV, craving is also a cardinal feature of sudden abstinence from nicotine use.

We hypothesise that respondents who are failed quitters in a telephone quitline report more severe symptoms compared with subjects who succeed in stopping smoking. This should hold even after controlling for potential confounders including age, gender, and use of nicotine replacement therapy (NRT). We also hypothesise that depression/depressed mood may be associated with failure to remain abstinent. The objective of this study was to describe the prevalence and intensity of different symptoms in relation to tobacco abstinence in a group of quitters calling a quitline. We also try to explore if there is a correlation between the different symptoms.

## Materials and methods

### Study population and setting

The study is based on a 12-month follow-up comprising all 1131 smokers who had called the Swedish quitline and signed up for smoking cessation treatment from February 2000 to November 2001. A total of 741 people (66%) reporting to have been abstinent for a minimum of 24 hours to a theoretical maximum of 12 months during the follow-up period were included in the study base. The same study population was used for all analyses.

The Swedish quitline [8,9] is a nationwide free of charge service that operates 51 hours per week through three to four telephone lines. All calls are registered on computerised patient records. All callers signing up for smoking cessation support by returning a registration form by mail are included.

Self reported point prevalence abstinence at 12 months was defined as "not a single puff of smoke during the last week".

### Measures

A questionnaire identifying 17 symptoms was sent by mail to all subjects 12–13 months after first contact with the quitline. Assessed symptoms are presented in Table 1 and nine correspond to the Diagnostic and Statistic Manual for Mental Disorders DSM-III-R [10]/DSM – IV criteria [7]. Increased appetite and weight gain were excluded because of the narrow time window used. Physiological symptoms not included in the DSM comprised nightmares, dizziness, mouth ulcers, sweating, muscular pain, cramps, constipation and other stomach trouble.

**Table 1:** Frequency and intensity of reported symptoms in smokers abstaining from tobacco at least 24 hours. (N = 741)

Symptoms	No		Low		Moderate		High	
	%	n	%	n	%	n	%	n
Craving	12%	(91)	15%	(115)	29%	(215)	43%	(320)
Irritability	24%	(177)	25%	(185)	27%	(203)	24%	(176)
Apprehension/Anxiety	27%	(200)	24%	(175)	25%	(182)	25%	(184)
Restlessness	18%	(135)	21%	(156)	27%	(199)	34%	(251)
Difficulties concentrating	29%	(216)	23%	(168)	24%	(181)	24%	(176)
Depression/depressed mood	35%	(261)	20%	(151)	21%	(153)	24%	(176)
Headache	61%	(450)	18%	(137)	13%	(94)	8%	(60)
Insomnia	46%	(341)	18%	(137)	15%	(111)	20%	(152)
Sleepiness/drowsiness	61%	(450)	17%	(128)	12%	(91)	10%	(72)
Nightmares	71%	(526/)	12%	(89)	9%	(64)	8%	(62)
Dizziness	66%	(493)	17%	(130)	9%	(64)	7%	(54)
Mouth ulcers	81%	(601)	7%	(55)	5%	(35)	7%	(50)
Sweating	58%	(429)	16%	(119)	14%	(107)	12%	(86)
Muscular pain	76%	(562)	12%	(90)	6%	(45)	6%	(44)
Cramps	88%	(650)	5%	(41)	4%	(27)	3%	(23)
Constipation	70%	(519)	12%	(89)	8%	(62)	10%	(71)
Other stomach trouble	75%	(558)	11%	(85)	8%	(56)	6%	(42)

The question used to assess symptoms was: "Did you experience any of the symptoms listed below when you abstained from smoking? – Reply to this question only if you have been abstinent for at least 24 h since your first contact with the quitline".

For self-assessment of symptoms we used: *none, low, moderate, and high *[11]. One additional question was asked to explore if those having depression/depressed mood as a symptom were more prone to relapse "Have you been unusually stressed or depressed during any period after your first contact with the quitline?".

NRT use was assessed by asking the question "which of the following nicotine replacement products did you use after your first contact with the quitline?". Pharmacological treatment included: chewing gum, transdermal nicotine patch, inhaler, nasal spray or tablets.

Ethical approval was obtained by Karolinska Institutet (Dnr 00-367).

### Statistical methods and presentation of data

Logistic regression analysis was used to calculate crude and adjusted odds ratios with 95% confidence interval. To analyse the relationship between symptoms and abstinence we dichotomised the response alternatives (none, low, moderate, and high) into low and high intensity. This was done so that for each variable, the two categories became as equally sized as possible. The cut-offs for the assessment of NRT use were "less than 5 weeks", "5 weeks or more", or "not at all". NRT was also assessed as "NRT" vs. "no NRT use". Significance levels for all tests were two-tailed. Statistical analysis was carried out using the Statistical Package for the Social sciences (SPSS 12.1).

A factor analysis approach where individual items aggregate into factors was used to explore the relationship between the different symptoms. Firstly, a principal component analysis was conducted to explore the correlation between the 17 symptoms. Secondly, varimax orthogonal rotation was done to simplify the interpretation of the factors by maximising the variance of the loadings within factors, across variables. It was decided a priori that the number of factors in the varimax rotation should be based on the number of Eigenvalues ≥ 1,0 in the principal component analysis and Cattell's scree test was performed [12-14]. Factor loadings greater than 0.35 was a priori considered to be of importance when interpreting the factors.

A factor loading matrix is created were correlations between factors and variable are presented. The first column is correlations between the first factor and each variable in turn. The second column is correlations between the second factor and each variable in turn and goes on the same way for the third factor. A factor is interpreted from the variables that are highly correlated with it – that have high loadings on it [14].

To assess the relationship between factors and abstinence, factor scores were calculated using the regression method and t-test was performed.

## Results

### Prevalence of symptoms

Of the 1131 subjects who agreed to participate in the study, 66% (741/1131) reported to have been smoke free for at least 24 hours. Of those 741, fourteen (2%) reported no symptoms (not in table). The description of all symptoms are given in Table 1. A total of 43% reported to have high craving and 34% reported high restlessness. Other relevant symptoms were reported by approximately one in four subjects: apprehension/anxiety 25%, irritability 24%, difficulties concentrating 24%, and depression/depressed mood 24% (Table 1).

### Abstinence

Of all subjects 48% (354/741) reported not a single puff of smoking during the previous week (point prevalence abstinence). There was no significant difference regarding the prevalence of symptoms when comparing those being smoke free for at least 24 hours with those being abstinent for seven days, two months or six months (not in table). The observed relationship between symptoms and abstinence remained even after adjustment for age, gender, and use of nicotine replacement therapy (not in table). Of those having depression/depressed mood 28% (184/649) reported abstinence compared with 72% (465/649) not being abstinent OR (1.4 – 1.8).

### NRT use

NRT was used by 80% (592/741). Of those reporting NRT use 34% (199/592) were smoke free compared to 27% (121/446) of subjects reporting not having used NRT, OR 1.4 (1.0–1.8), (not in table). Of people using NRT for 5 weeks or longer 48% (115/237) reported to be smoke free, compared to 27% (121/446) in those who did not use NRT, OR 2.5 (1.8–3.5), (not in table). When the analysis was restricted to include only people reporting having been smoke free for at least 5 weeks, those using NRT were less likely to be point-prevalence smoke free 54% (171/317) vs. 65% (121/186) OR 0.6 (0.4–0.9), (not in table).

High intensity of symptoms related to unsuccessful quitting attempts and comprised craving, irritability, apprehension/anxiety, difficulties concentrating, restlessness, depression/depressed mood and insomnia. A reverse relation was observed for mouth ulcers (Table 2).

**Table 2:** Withdrawal symptoms versus abstinence. Presenting percentage and proportion reporting to be abstinent. (N = 741)

Symptoms	Low intensity	High intensity^(ref)^	OR 95%CI^#^
Craving	49% (207/421)	32% (103/320)	2.0 1.5–2.8*
Irritability	45% (163/362)	39% (147/379)	1.3 1.0–1.7*
Apprehension/Anxiety	47% (177/375)	36% (133/366)	1.6 1.2–2.1*
Restlessness	49% (142/291)	37% (168/450)	1.6 1.2–2.2*
Difficulties concentrating	46% (175/384)	38% (135/357)	1.4 1.0–1.8*
Depression/depressed mood	48% (196/412)	35% (114/329)	1.7 1.3–2.3*
Headache	40% (182/450)	44% (128/291)	0.9 0.6–1.2
Insomnia	45% (155/341)	39% (155/400)	1.3 1.0–1.8*
Sleepiness/drowsiness	41% (186/450)	43% (124/291)	0.9 0.7–1.3
Nightmares	42% (222/526)	41% (88/215)	1.1 0.8–1.5
Dizziness	42% (206/493)	42% (104/248)	1.0 0.7–1.4
Mouth ulcers	40% (238/601)	51% (72/140)	0.6 0.4–0.9*
Sweating	42% (180/429)	42% (130/312)	1.0 0.8–1.4
Muscular pain	41% (233/562)	43% (77/179)	0.9 0.7–1.3
Cramps	41% (269/650)	45% (41/91)	0.9 0.6–1.3
Constipation	43% (222/519)	40% (88/222)	1.1 0.8–1.6
Other stomach trouble	41% (227/558)	45% (83/183)	0.8 0.6–1.2

Note: Dichotomisation was done so that for each symptom, the two categories became as equally sized as possible

* Difference statistically significant

# Crude odds ratios with 95% confidence interval

### Factor analysis

Three factors whose eigenvalues (i.e., the sum of the squared factor loadings) were greater than 1 were identified and accounted for 49% of the variance. Factor loading greater than 0.35 was considered in the interpretation of the factors (Table 3). The greater the loading, the more the variable is considered a pure measure for the factor [15]. *Factor 1 *(*psychological*) comprised symptoms that were mainly psychological and to some extent neurological in nature. Assigned to this group were craving, irritability, apprehension/anxiety, difficulties concentrating (also included in factor 3), restlessness, and depression/depressed mood (also included in factor 3), (Table 3). *Factor 2 *(*physiological*) included symptoms that were primarily physical (somatic) and partly neurological including mouth ulcers, dizziness, (also included in factor 3) sweating, muscular pain, cramps, constipation, and other stomach trouble (Table 3). *Factor 3 *(*neurological*) comprised mainly symptoms that may be termed neurological as well as some symptoms that are more psychological in nature including headache, insomnia, sleepiness/drowsiness, nightmares, dizziness, difficulties concentrating (also included in factor 1), and depression/depressed mood (also included in factor 1), (Table 3).

**Table 3:** Factor analysis including items of distinctive groups, psychological, physiological and neurological.

	Factor		
	
	1	2	3
Craving	**.701**	.176	-.238
Irritability	**.714**	.090	.175
Apprehension/anxiety	**.741**	.045	.328
Restlessness	**.738**	.085	.220
Difficulties concentrating	**.713**	.085	**.367**
Depression/depressed mood	**.546**	.046	**.444**
Headache	.182	.192	**.647**
Insomnia	.257	.195	**.625**
Sleepiness/drowsiness	.136	.209	**.633**
Nightmares	.155	.274	**.576**
Dizziness	.107	**.453**	**.432**
Mouth ulcers	.056	**.583**	.080
Sweating	.209	**.575**	.317
Muscular pain	.047	**.693**	.151
Cramps	.053	**.726**	.017
Constipation	.093	**.533**	.205
Other stomach trouble	.037	**.526**	.276

Note: Factor loadings greater than 0.35 are boldface and constitute a relationship defining a factor

### Factor scores and abstinence

The comparison between the mean values of the factor scores revealed that a high factor score on factor 1 (psychological) was significantly related to unsuccessful quitting attempts.

### NRT and symptoms

**Figure 1:**
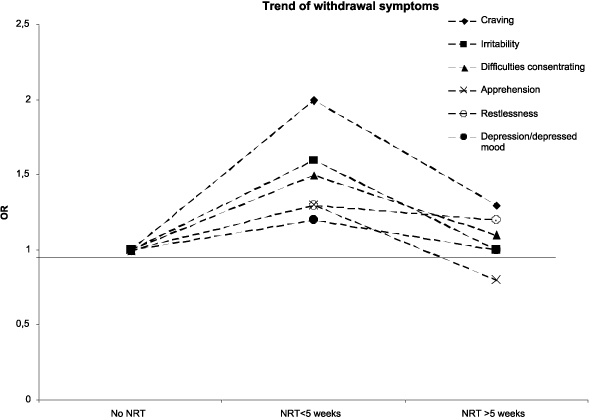
**Symptoms in relation to NRT use**. Presenting odds ratios for high intensity of different symptoms in relation to NRT use.

When compared with those reporting *not *having used nicotine replacement therapy (NRT), using NRT for less than 5 weeks was significantly correlated with an increased prevalence of craving, irritability, difficulties concentrating and mouth ulcers, and a decreased prevalence of cramps (Table 4). With exception of mouth ulcers these correlations did not remain when comparing non-users of NRT to users of NRT for 5 weeks or longer. The tendency of increased prevalence for users of NRT for less than 5 weeks, but not for users of NRT for 5 weeks or longer, was seen for all symptoms comprising the psychological factor (Figure 1). Constipation and other stomach trouble showed yet another pattern with an increase of prevalence for users of NRT for 5 weeks or longer but not for users of NRT for less than 5 weeks (Table 4). Of all NRT users those using chewing gum reported more ulceration compared with those using non-oral NRT, 24% (53/216) versus 18% (40/227), OR 1.5 (1.0–2.4) (not in table).

**Table 4:** How NRT is related to different symptoms N = 625

Symptoms	Ref No NRT^#^	NRT<5 weeks	NRT ≥ 5 weeks
Craving	36% (88/242)	53% (98/185)	42% (83/198)
		2.0 (1.3–2.9)*	1,3 (0.9–1.9)
Irritability	48% (117/242)	60% (111/185)	47% (94/198)
		1.6 (1.1–2.4)*	1.0 (0.7–1.4)
Apprehension/anxiety	51% (123/242)	57% (106/185)	45% (89/198)
		1.3 (0.9–1.9)	0.8 (0.5–1.2)
Restlessness	56% (136/242)	63% (116/185)	61% (120/198)
		1.3 (0.9–1.9)	1.2 (0.8–1.8)
Difficulties concentrating	44% (107/242)	55% (101/185)	46% (92/198)
		1.5 (1.0–2.2)*	1.1 (0.8–1.6)
Depression/depressed mood	43% (103/242)	47% (87/185)	41% (82/198)
		1.2 (0.8–1.8)	1.0 (0.7–1.4)
Headache	38% (91/242)	43% (80/185)	38% (76/198)
		1.3 (0.9–1.9)	1.0 (0.7–1.5)
Insomnia	49% (118/242)	54% (99/185)	56% (111/198)
		1.2 (0.8–1.8)	1.3 (0.9–2.0)
Sleepiness/drowsiness	39% (94/242)	41% (76/185)	39% (78/198)
		1.1 (0.7–1.6)	1.0 (0.7–1.5)
Nightmares	28% (67/242)	27% (50/185)	32% (64/198)
		1.0 (0.6–1.5)	1.2 (0.8–1.9)
Dizziness	35% (85/242)	35% (64185)	32% (64/198)
		1.0 (0.7–1.5)	0.9 (0.6–1.3)
Mouth ulcers	15% (36/242)	22% (41/185)	24% (47/198)
		1.6 (1.0–2.7)*	1.8 (1.1–2.9) *
Sweating	40% (96/242)	45% (83/185)	41% (82/198)
		1.2 (0.8–1.8)	1.1 (0.7–1.6)
Muscular pain	26% (62/242)	23% (43/185)	23% (46/198)
		0.9 (0.6–1.4)	0.9 (0.6–1.4)
Cramps	15% (35/242)	6% (11/185)	15% (29/198)
		0.4 (0.2–0.8)*	1.0 (0.6–1.7)
Constipation	26% (62/242)	29% (53/185)	35% (69/198)
		1.2 (0.8–1.8)	1.6 (1.0–2.3) *
Other stomach trouble	21% (52/242)	25% (47/185)	29% (58/198)
		1.2 (0.8–1.9)	1.5 (1.0–2.3) *

Note: Dichotomisation was done so that for each symptom, the two categories became as equally sized as possible

# Crude odds ratios with 95% confidence interval comparing with no NRT use

*Difference statistically significant

## Discussion

Three factors of symptoms reported to be related to tobacco abstinence were identified among this group of consecutive callers to the Swedish quitline (Table 3): *"psychological" *(factor 1), *"physiological" *(factor 2), and *"neurological" *(factor 3). The symptoms that were most frequently reported (Table 1) were the symptoms comprising what we labelled as the psychological factor. All symptoms comprising the psychological factor were statistically related to point prevalence abstinence at 12 months, with those reporting low intensity of the symptoms being more likely to report abstinence.

### Symptoms and abstinence

All symptoms comprising the psychological factor were negatively related to point prevalence abstinence at 12 months. This factor comprised largely the same symptoms that have previously been highlighted in the assessment of tobacco cessation in other settings [16,17] and related to abstinence [18-20]. Three prospective studies have reported depression/depressed mood as being predictive of unsuccessful smoking cessation and relapse [21,20,22]. This is in line with our results where having depression/depressed mood as a symptom proved to be associated with failure to remain abstinent. Also in table 2 having high intensity of depression/depressed mood related negatively to abstinence. (Table 2).

In our study, only two symptoms not belonging to the psychological factor, were related to abstinence: insomnia and mouth ulcers. Insomnia included in the neurological factor (factor 3) in Table 3 was one of three symptoms assessing sleeping disorders that comprised half of the symptoms in this factor. Studies of nicotine withdrawal have consistently found sleep disturbance to be a common complaint [23] and since three of the symptoms insomnia, sleepiness/drowsiness and nightmares related to sleep disturbance and had relatively high factor loadings we might have a latent dimension of sleeping disorders in factor 3.

The other symptom not belonging to the psychological factor, and significantly related to abstinence was mouth ulcers, belonging to the physiological factor (factor 2) in Table 3. In this case the relationship was reverse. Subjects reporting mouth ulcers were more likely to be abstinent. This might be explained by the fact that NRT use was positively related to mouth ulcers. In our study, high intensity of mouth ulcers was reported by 7%. Another study found that 8% reported severe mouth ulcers [24] in relation to smoking cessation.

It has been reported that one in ten patients has severe problems with constipation in relation to smoking cessation [25]. This was confirmed in our results.

### Symptoms and NRT

All psychological symptoms appeared to be associated with NRT use. People using NRT for less then 5 weeks reported a higher prevalence of these symptoms compared with individuals not using NRT and those who used NRT for 5 weeks or longer.

It would be expected that people experiencing low intensity of these symptoms would be less prone to use NRT. Figure 1 illustrates that people using NRT to ease psychological symptoms need taking higher doses to achieve results. These findings are in line with previous reports [26] and present recommendations regarding NRT [27]. There was a significant increase of mouth ulcers in NRT users compared with non-users. One hypothesis is that this may be due to a changed balance in oral microbial flora secondary to the toxic effect of nicotine.

### NRT and abstinence

The hypothesis that nicotine withdrawal may be an important reason why individuals persist in their smoking behaviour and are relatively unsuccessful in their smoking cessation attempts [28,29] is supported by our results. That can be seen by the relationship between low prevalence of symptoms comprising the psychological factor, increased abstinence and the suppressing effect of NRT use for 5 weeks or longer on these symptoms.

However, the relationship between nicotine withdrawal and unsuccessful smoking cessation has been ambiguous in previous reviews of the literature [30]. One reason for this unclear relationship may be insufficient control for psychological and environmental factors strongly related to abstinence [8]. These psychological and environmental factors will delude the relationship between nicotine dependency and successful cessation if not controlled for. Also, the assessment of dependence usually comprises questionnaires that should at best be viewed as indicators of dependence and are by no means objective measures of a physiological phenomenon [31]. There is a possibility of unidentified physiological factors of dependence in tobacco that may partly be responsible for the diffuse relationship between nicotine withdrawal and unsuccessful smoking cessation. However, such a hypothesis is highly speculative. However, the risk of experiencing nicotine withdrawal after a quit attempt is known to be partly related to genetic vulnerability [18].

Gender has been reported to influence withdrawal discomfort [32]. However, other studies, including ours, found no differences in overall withdrawal severity between men and women [17,23,32].

### Methodological limitations

The present study assesses a population of smokers having called a quitline for smoking cessation support. We do not know if those not participating in the follow-up (30%) had more withdrawal symptoms. However, in a previously published study [33] we assessed the behaviour of non-responders by using telephone interviews with a sample of people not participating in the 12 months follow-up. No significant difference regarding abstinence rates between responders and non-responders was found in our material.

Another possible source of error includes the use of single item measures of symptoms, rating each symptom on a four point prevalence intensity scale [34,35]. An alternative would have been to use validated psychometric scales to assess the prevalence and intensity of symptoms like anxiety and depression/depressed mood. This was not deemed practical in the quitline setting due to the large number of patients.

As most smokers report multiple quit attempts before they succeed [36] it is plausible that individuals eventually learn to endure the symptoms of withdrawal. Thus, the reported symptoms (Table 1) may be an underestimation. Also, coping strategies such as relaxation or seeking social support which may have been achieved through the quitline counsellors, may have moderated symptom stress. Another caveat in a retrospective study is recall bias.

The questionnaire used by the Swedish quitline at the time assessed withdrawal symptoms only among smokers who had made a serious attempt to quit and had been totally tobacco free for at least 24 hours. Thus, comparing symptoms between smokers not attempting to quit and those who were at least temporary successful (24 hours or more) was not possible. Further, we do not know the prevalence and intensity of the assessed symptoms in the general population of non smokers. Thus, baseline data on the prevalence of the symptoms was not available which is a problem for the presentation of symptom prevalence in Table 1. However, this is probably not a major problem for the analysis of the relationship between symptoms and to what extent the reported symptoms were related to the 12 months abstinence rate.

It has been argued that a quit attempt that lasted a week or longer in the last year appears less biased by recall than any attempt of a day or longer in the last year [37]. However, the 24 hour time frame of abstinence used for symptom assessment was selected to achieve better statistical power in the analysis. It may be argued that the 24 hour time window is too narrow but we argue that this is justifiable since there was no significant difference detected regarding prevalence of symptoms in the 24 hour group when compared with people reporting longer abstinence (seven days, two and 6 months). It was noted [1] that self-report ratings of behaviours and internal states becomes less valid when participants are asked to rate them over a substantial period of time (i.e. 2–4 weeks) rather than a shorter time period (i.e. within the past 24 hrs.).

Unfortunately only abstinent subjects were examined since the question only addressed those being abstinent for at least 24 h. This may have understated symptom severity.

NRT was measured only with a question assessing duration of treatment and the cut-offs were less than 5 weeks, 5 weeks or more, or not at all. There was no measure of the intensity of the NRT treatment during the assessed time frame. This is likely to reduce the sensitivity of assessment of the impact NRT may have on the severity of the symptoms.

Although the 17 symptoms measured here fell almost cleanly into three distinct groupings, this does not mean that there are only three factors of symptoms related to tobacco withdrawal. There may be other factors not captured by these 17 items. Additional studies need to be done to further explore the mechanisms that produce the three factors observed in this study.

In conclusion, our results indicate that high prevalence of symptoms comprising the psychological factor relate to unsuccessful quitting attempts in a population-based setting. NRT appeared to have a positive effect on relieving these symptoms when used for five weeks or more.

## Competing interests

The authors declare that they have no competing interests.
